# Can antimicrobial photodynamic therapy serve as an effective adjunct protocol for disinfecting the necrotic root canal system? A randomized controlled study

**DOI:** 10.1038/s41405-024-00239-y

**Published:** 2024-06-20

**Authors:** Remy Barazy, Hisham Alafif, Hassan Achour, Ahmad Al-Aloul, Yasser Alsayed Tolibah

**Affiliations:** 1 Department of Endodontics, Faculty of Dentistry, Damascus University, Damascus, Syrian Arab Republic; 2 Department of Endodontics, Faculty of Dentistry, Al-Sham Private University, Damascus, Syrian Arab Republic; 3 Department of Pediatric Dentistry, Faculty of Dentistry, Damascus University, Damascus, Syrian Arab Republic

**Keywords:** Oral diseases, Oral microbiology

## Abstract

**Background:**

Bacterial infection plays an important role in persistent periapical lesions and inadequate disinfection of root canals is considered the biggest factor responsible for endodontic treatment failure. Antimicrobial Photodynamic Therapy (aPDT) has become the latest choice to eradicate microorganisms in root canals.

**Objective:**

This study aims to evaluate the effectiveness of Antimicrobial Photodynamic Therapy (aPDT) in bacterial count reduction compared to Passive Ultrasonic Activation (PUI) and Ca(OH)2 dressings.

**Materials and methods:**

Forty-five anterior single canal teeth with medium-sized periapical lesions (2–5 mm) were divided into three groups according to the disinfecting technique (each group consists of 15 canals with 1:1:1 allocation ratio): Group A: Ca(OH)2 dressing. Group B: Passive Ultrasonic Activation (PUI). Group C: Antimicrobial Photodynamic Therapy (aPDT). Direct bacterial viable count method was used to count the colonies forming units (CFU) before and after the disinfecting and the bacterial count reduction was estimated, the statistical analysis was performed at a 95% confidence level using the Chi-square and Mann–Whitney *U* test.

**Results:**

aPDT showed no statistically significant difference when compared to passive ultrasonic irrigation (*P* > 0.05) but showed higher and more promising results when compared to Ca(OH)2 dressings (*P* < 0.05).

**Conclusions:**

aPDT has the ability and effectiveness as a disinfecting technique in necrotic and infected root canals.

**Clinical significance of the study:**

The results of this clinical trial provide that aPDT can be considered an adjunct method for root canal disinfection with the same effectiveness as passive ultrasonic irrigation.

## Introduction

The main goal of performing endodontic treatment in infected root canals is to promote and accelerate the healing process of apical periodontitis. That involves the removal of pulp, dentine debris, bacteria, and toxins [1].

The variety in bacterial flora, viruses, fungi, and archaea among different types of root canal infections, and the development of antibiotic resistance have caused variable responses to traditional treatments such as triple antibiotic and calcium hydroxide paste dressings [2]. To improve the success rates of endodontic treatment, especially in cases with periapical lesions, several modern techniques have been developed to reduce bacteria in root canals, including Antimicrobial Photodynamic Therapy (aPDT) [3].

aPDT is defined as “light-induced inactivation of cells, microorganisms, or molecules” [4]. This technique has been recently used to improve endodontic bacterial disinfection either as an adjunct technique to conventional chemo-mechanical procedures or in combination with other disinfecting techniques, particularly in cases treated or retreated in single visits [5]. aPDT is applied in two stages: The first step is to apply the photosensitizer agent (PS) on the target tissue for a period called pre-irradiation time. The second step is to expose this agent to visible light at an appropriate wavelength that is compatible with the photosensitizer in the presence of oxygen, either directly on target tissues or using special endodontics tips to deliver light to the apical third of the canal inducing specific bacterial cell damage [6].

There are two ways PS can react with bacterial cells. The type I reaction, or electron transfer reaction, results in producing reactive oxygen species (ROS) such as hydrogen peroxide, hydroxyl radicals, and superoxide. This reaction causes irreversible cell damage which can involve leakage of cellular contents or inactivation of membrane transport systems and enzymes [7]. As for the type II reaction, PS reaches a triple excited state and produces a highly reactive oxygen or singlet oxygen (^1^O_2_) molecule, which leads to bacterial cell death by oxidative damage affecting their plasma membrane, including proteins, lipids, and DNA, without compromising host cell viability [8].

In endodontic clinical applications, recent studies have supported the use of photosensitizers that fall within the blue or red absorption spectrum as effective candidates for producing highly reactive oxygen species [9]. Blue light devices with a wavelength of 450–470 nm were used with an output power ranging between 4000–7500 mW/cm^2^ [10], while red light devices with a wavelength of 630–660 nm were used with an output power ranging between 2000–4000 mW/cm^2^ [11].

Various photosensitizers consisting of natural dyes such as curcumin or synthetic dyes such as methylene blue, toluidine red, and others were used [12]. Synthetic dyes are considered more stable than natural dyes, but natural dyes cause fewer side effects and do not interfere with the action of intracanal antibiotic dressings used in endodontic treatments [13]. Moreover, this technique could be an alternative to intracanal antibiotic dressings, avoiding possible antibiotic resistance [3].

New photosensitizers have been developed with new formulations of a mixture of natural and synthetic dyes, also containing an oxidant and oxygen transporter that increases the effectiveness of the agent in destroying intratubular bacterial biofilms [14]. An example of these new photosensitizer is QROXB2. It contains Curcumin, Riboflavin (vitamin B2), and hydrogen peroxide as an oxidant agent [15].

Several studies have been conducted using different light sources and different photosensitizers, but these studies obtained varying results even when the light and photosensitizer used were standardized, as a result of using different concentrations of photosensitizer, different photo-activation periods, and different output powers of the light source, which makes a comparison between these studies difficult [16–19].

It is well-documented in the medical literature that the alkalinity of calcium hydroxide neutralizes the acidity in inflamed areas by counteracting the acidic metabolic byproducts produced by phagocytic cells and osteoclasts. This neutralization halts the dissolution of hard tissues and indirectly reduces inflammation by decreasing periapical exudation. Additionally, calcium hydroxide exhibits antimicrobial properties against pathogens causing pulpitis due to its high alkalinity, which results from its rapid dissolution in aqueous solution, thereby releasing hydroxyl ions that act as strong oxidizing agents [20, 21].

Passive ultrasonic irrigation (PUI) offers several benefits: it improves the removal of pulp and dentin debris, is more efficient at eliminating bacteria compared to manual agitation, and effectively cleans curved canals and isthmuses. PUI works by transmitting acoustic energy via ultrasonic waves through a file or oscillating wire to the irrigation solution, resulting in two main actions [22]. The first action, acoustic streaming, involves generating hydrodynamic force and dynamic pressure within the irrigant, while the second action, cavitation, involves the formation of vapor-containing bubbles produced by ultrasonic waves within a fluid [23].

There are no previous studies that used FlashMax P7(CMS Dental, Denmark) device and its photosensitizer to reduce the bacterial count in necrotic root canals, so the results were discussed with studies that used aPDT with other light sources and photosensitizers or separated components of QROXB2. So, this study aimed to evaluate the antibacterial activity of aPDT using FlashMax P7 and QROXB2 in comparison with PUI and Calcium hydroxide Ca(OH)_2_ dressings in necrotic root canals.

## Materials and methods

### Study design, settings, and ethical approval

This single‐blinded randomized controlled trial used a three‐arm parallel superiority design with a 1:1:1 allocation ratio undertaken from March 2020 to March 2023 at the Endodontic Department of the Faculty of Dentistry, Damascus University, Damascus, Syria. This study adhered to the ethical guidelines of the Declaration of Helsinki and received ethical approval from the Local Research Ethics Committee of the Faculty of Dentistry (Approval No. UDDS‐222‐28202022/SRC‐1976).

### Sample size calculation

Based on the data of a previous study [24], the sample size in the present study was calculated by G* Power 3.1.9.4 (Heinrich-Heine-Universität, Düsseldorf, Germany). In the ANOVA study, sample sizes of 15, 15, and 15 were obtained from the 3 groups. The total sample of 45 subjects achieves 0.55 effect size f and 90% power to detect differences with a 0.05 significance level.

### Randomization

The randomization process involved the use of cards detailing distinct disinfection protocols, which were enclosed in opaque, sealed envelopes and then randomly numbered using an Excel spreadsheet’s random allocation function (Microsoft, Washington, USA). Patients were directed to select an envelope at random, leading to their assignment into one of three groups: Group 1, comprising 15 canals subjected to aPDT as a disinfection protocol; Group 2, consisting of 15 canals treated with passive ultrasonic irrigation; and Group 3, with 15 canals undergoing Ca(OH)2 dressing as a disinfection protocol.

### Blinding

The treating clinician and the patient could not be blinded in this study, as there is a physical difference between different disinfecting techniques which is clear to the dentist providing the treatment. However, the outcomes assessors who assessed the bacterial count reduction in petri dishes were blinded to the disinfection protocol used.

### Recruitment and eligibility criteria

The recruited participants involved patients aged between 18 and 45 years who have medium-sized periapical lesions (3–5) mm [25] located around the apex of anterior single canal teeth who attended the endodontic department during the study period. Patients were excluded from the study if they had systemic diseases that compromised their general immune status, teeth were previously treated, had internal or external root resorption, periodontitis, or when the patient had edema, pus, or severe pain. Patients who agreed to participate in the study were given all the information regarding the study and signed informed consent before treatment.

### Clinical procedures

Periapical radiographs were taken using a special sensor positioner (Vatech, Hwaseong, Korea) to study the case (tooth anatomy, size of periapical lesion in millimeters, and curvature of canal) to determine included teeth. After identifying the enrolled patients, they were instructed to rinse their mouths with a 0.12% chlorhexidine solution (Aphamia, Hamah, Syria) prior to commencing the procedure to reduce the viable microbial content of dental aerosols [26]. Subsequently, under local anesthesia (Huons Lidocaine HCL, Seoul, Korea) and rubber dam isolation (Sanctuary, Perak, Malaysia), work area and teeth were disinfected with 30% hydrogen peroxide and 10% iodine to reduce the risk of contamination while taking the swabs from the canal [24]. Afterward, caries was removed and the access cavity was opened using an Endo-Z bur (Dentsply Maillefer, Tusla, Oklahoma, USA). Subsequently, canals were filled with saline solution, then a sterile #15 hedströem file (Mani, Tochigi, Japan) in an in-and-out motion along the root canal dentine was done. Afterward, a sterile paper point (Gabadent, Guangdong, China) was inserted into the canal for 60 s. The canal drying procedure was repeated 3 times, where the last paper point was placed in Sterilized tightly sealed glass test tube of 5 ml capacity, containing sterile saline solution. This sample was called Canal Fluid Sample N0 (CFS0). The CFS0 collecting method was similar to the described by Kharsa and colleagues [24].

Root canal working length was determined using a K-file of appropriate size and electronic apex locator (E-PEX, Eighteeth, Changzhou, China), then canals were prepared using Fanta F One files (Fanta, China), where the final apical preparation size was (25.04) to (40.04) concerning the initial size of apex, and irrigated with 5 ml 5.25% sodium hypochlorite and saline solution [27].

### Afterward, samples were randomly divided into three groups according to the disinfecting technique applied to root canal

Group A: Ca(OH)2 dressing (Enpunuo Biotechnology, Changsha, China) was applied in the root canal. 7 days later, Ca(OH)2 dressing was removed. Afterward, each canal was irrigated with 15 mL of EDTA, instrumented with its final apical file, irrigated with 20 ml of 5.25% sodium hypochlorite to remove calcium hydroxide dressing debris [28], and then irrigated with 4 ml of 5% sodium thiosulfate to neutralize the sodium hypochlorite [29].

Group B: Passive Ultrasonic Irrigation (PUI) was performed for root canal disinfection, 3 ml of 5.25% sodium hypochlorite was applied followed by ultrasonic activation (Ultra X, Eighteeth, Changzhou, China). PUI was conducted with intermittent flush (3 cycles x 20 s of ultrasonic activation) [30]. Afterward, canals were irrigated with 4 ml of 5% sodium thiosulfate to neutralize the sodium hypochlorite [31].

**Fig. 1:**
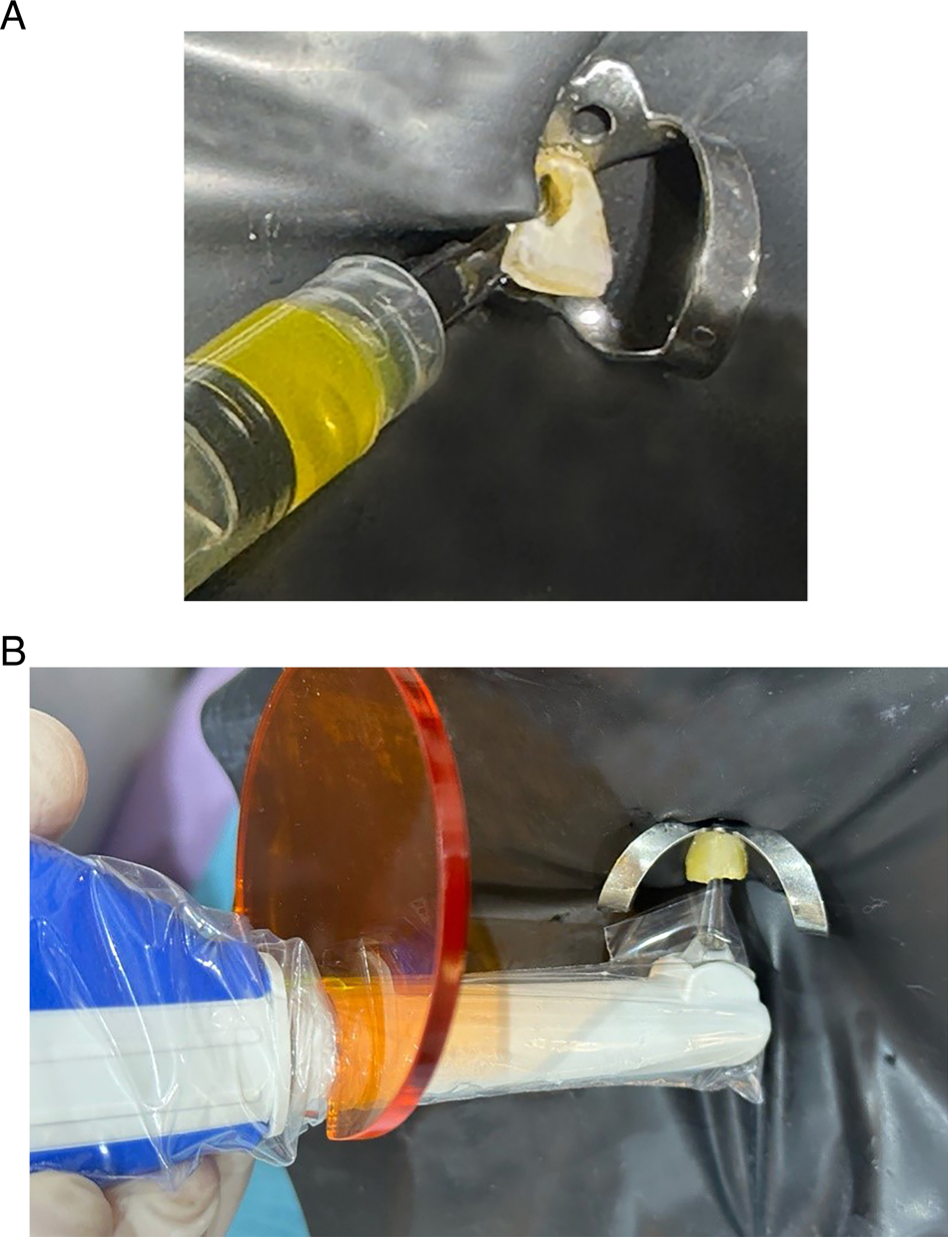
A clinical case of using the Flash Max P7 device and QROXB2 photosensitizer for canal sterilization. aPDT therapy steps: **A** applying QROXB2 photosensitizer into root canal, and **B** photo activation for 30 s.

Group C: aPDT was performed for root canal disinfecting using Flash Max P7 device (CMS Dental, Denmark), with 460 wavelength blue light and 7500 mW/cm2. QROXB2 photosensitizer of the FlashMax P7 device was prepared by drawing 0.5 ml of distilled water into a syringe containing photosensitizer and shaking it for 3 min until the powder was completely dissolved in water. Root canal was then immersed with photosensitizer and agitated by a #15 K-file for three minutes (pre irradiation time). Then the light transmitting head (endo tip) was inserted to two-thirds of working length of studied canal and FlashMax P7 was activated for 30 s (Fig. 1). Finally, canals in this group received copious irrigation with 20 ml of distilled water to remove the QROXB2 photosensitizer debris of the canal.

After disinfection protocols was applied, sample was collected in the same previously mentioned method. This sample was called Canal Fluid Sample N1 (CFS1). The CFS1 collecting method was similar to the described by Kharsa and colleagues [24].

The final irrigation sequence was 5 mL of 17% ethylenediaminetetraacetic acid (EDTA) for 3 min, followed by 5 mL of 5.25% NaOCl and 5 mL saline solution [31]. Then canals were dried with paper points and filled with gutta-percha and AH plus sealer using warm vertical obturation with Fast Pack and Fast Fill devices (Eighteeth, Changzhou, China) [32].

### Bacterial culturing

**Fig. 2:**
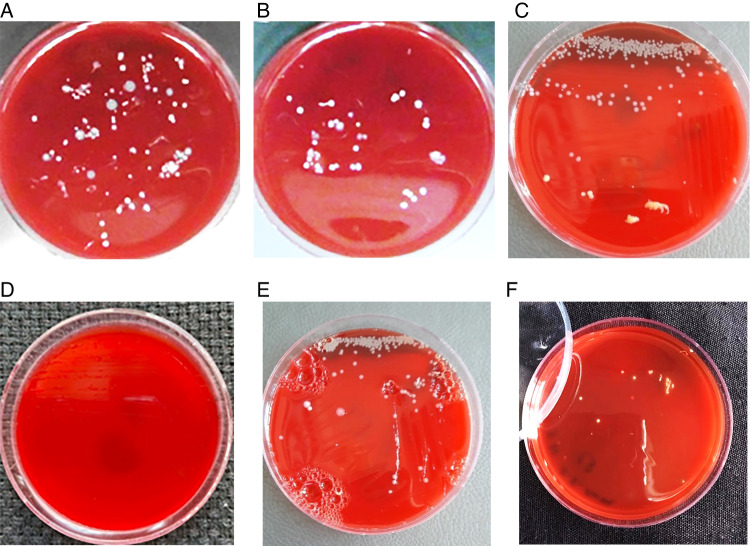
Photographs of Petri dishes for aerobic incubation of bacteria of swabs taken from included necrotic root canals. Bacterial culture after 24 h incubation in aerobic conditions: **A** CFU 0 Ca(OH)2 group, **B** CFU 1 Ca(OH)2 group, **C** CFU 0 PUI group, **D** CFU 1 PUI group, **E** CFU 0 aPDT group, and **F** CFU 1 aPDT group.

**Fig. 3:**
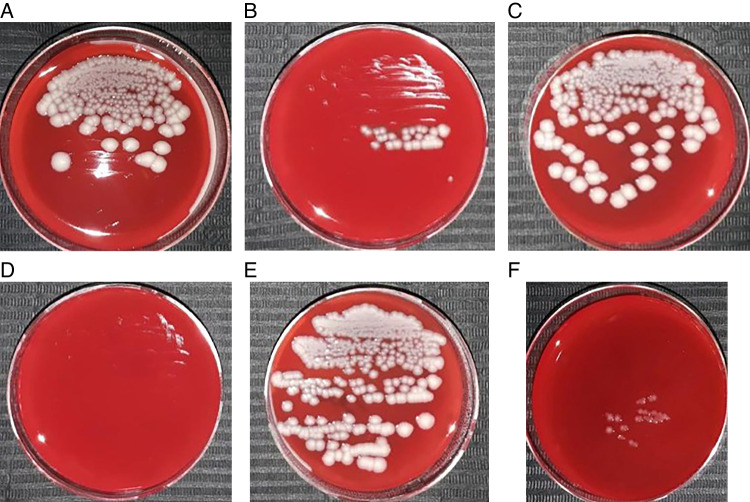
Photographs of Petri dishes for anaerobic incubation of bacteria of swabs taken from included necrotic root canals. Bacterial culture after 24 h incubation in anaerobic conditions: **A** CFU 0 Ca(OH)2 group, **B** CFU 1 Ca(OH)2 group, **C** CFU 0 PUI group, **D** CFU 1 PUI group, **E** CFU 0 aPDT group, and **F** CFU 1 aPDT group.

CFS0 and CFS1 glass test tube samples were placed on a vortex device (CSL-VORTEX, Thistle Scientific Ltd, Glasgow, United Kingdom) for 30 s to obtain a homogeneous bacterial suspension. Afterward, the bacterial culturing process was carried out by taking 100 μl samples from the CFS0 and CFS1 bacterial suspension using a micropipette on a blood agar medium after performing a series of dilutions. Dishes were placed in an incubator (Bacteriological Incubator 6640-01-071- 6596/National Appliance Heinicke Co. Tualatin, USA) according to the conditions for aerobic culture and anaerobic culture at 37 °C for 48 h. Colonies Forming Units (CFU) were counted in samples, where each canal has four petri dishes (two dishes for CFS0 and CFS1 in the aerobic culture (Fig. 2) and two dishes for CFS0 and CFS1 in the anaerobic culture) (Fig. 3).

### Subsequently, the vital count of bacterial colony forming units (CFU) in (1 ml) was calculated using the following equation

Number of bacteria/ml = number of CFU counted x Reciprocal of dilution factor x 10 (where the dilution factor adopted in counting dishes was 10^-2^).

The bacteria units’ number was converted into logarithmic numbers to facilitate bacterial colonies counting and statistical analysis.

### Statistical analysis

The collected data were tabulated and analyzed using SPSS software (SPSS Version 20, IBM SPSS Inc., Chicago, IL, U). The Shapiro–Wilk reveled that the quantitative measurements showed abnormal distribution, therefore the Kruskal–Wallis and Mann–Whitney *U* tests was used to assess the mean logarithm of bacterial reduction between groups. The level of significance was set at *a* < 0.05.

## Results

A total of 45 anterior teeth with medium sized periapical lesions in 45 patients were included in the study. No significant differences were reported between the groups regarding the age (*P* > 0.05) and gender (*P* > 0.05) of the treated patients.

Table 1 summarizes the evaluation of decimal logarithm of the bacterial colony counts among the groups of the disinfection protocols according to the stage studied. Kruskal–Wallis test shows significant difference between at least two groups after applying disinfecting protocol in aerobic and anaerobic conditions.

**Table 1:** Descriptive statistics of the decimal logarithm of the bacterial colony counts among the groups in aerobic and anaerobic conditions and the *P*-values of significance testing.

Condition	Stage	Disinfection Protocol group	Number	Mean	Standard Deviation	Minimum	Maximum	Chi-square value	*P-value
Aerobic	Before applying disinfecting protocol (CFS0)	Ca(OH)_2_ dressing	15	220.60	68.07	120	336	0.296	0.862
		PUI	15	231.80	52.14	165	324		
		aPDT	15	230.33	65.95	105	324		
	After applying disinfecting protocol (CFS1)	Ca(OH)_2_ dressing	15	22.80	15.51	0	49	10.116	0.006**
		PUI	15	4.97	6.60	0	16		
		aPDT	15	10	7.45	0	23		
Anaerobic	Before applying disinfecting protocol (CFS0)	Ca(OH)_2_ dressing	15	366.47	104.03	200	480	0.377	0.828
		PUI	15	378.33	60.54	275	483		
		aPDT	15	352.73	95.27	175	510		
	After applying disinfecting protocol (CFS1)	Ca(OH)_2_ dressing	15	35.13	16.00	10	64	16.054	0.000**
		PUI	15	14.13	8.13	5	30		
		aPDT	15	15.53	9.88	2	30		

*Kruskal–Wallis test, **Significant differences.

Table 2 summarizes the Mann–Whitney *U* test results conducted to assess the significance of the pairwise differences in the decimal logarithm values of the bacterial colony counts between each two groups of the disinfection protocols after aerobic bacteria incubation and anaerobic conditions in the research sample.

**Table 2:** Descriptive statistics of pairwise comparisons between the groups and the *P*-values of significance testing.

Condition	Stage	Group	Group	U value	*P value
Aerobic	After disinfection (CFS1)	aPDT	PUI	83.0	0.219
			Ca(OH)_2_	56.5	**0.020
		PUI	Ca(OH)_2_	43.5	**0.004
Anaerobic	After disinfection (CFS1)	aPDT	PUI	110.5	0.934
			Ca(OH)_2_	34.5	**0.001
		PUI	Ca(OH)_2_	24.5	**0.000

*Mann–Whitney *U* test, **Significant differences.

## Discussion

Endodontic treatment depends on the ability of each step to eliminate infection when the microbial nature of periapical periodontitis is taken into account [33]. Complete disinfection followed by a good canal obturation allows teeth affected by periapical periodontitis to heal [34].

A search in the literature showed that this clinical study is the first one to compare three types of disinfecting protocols (Ca(OH)_2_ dressings, PUI, and aPDT) in bacterial count reduction in patients with necrotic root canals in anterior single canal teeth.

To standardize the specimens and control anatomical differences, anterior teeth were selected due to their relatively straight root canal anatomy, minimizing variations in canal morphology, where the microbiota may be different in the different canals of multirooted teeth [35].

The FlashMax P7 (CMS Dental, Denmark) was selected as the device for the aPDT technique due to its manufacturer’s advocacy as “Number 1 in biofilm control.” Additionally, the QROXB2 photosensitizer, which contains curcumin, was chosen for its safety profile for patients and its demonstrated efficacy against oral biofilm [36].

The conversion of sound energy into heat through ultrasonic activation results in the elevated temperature of the irrigant, thereby boosting its effectiveness in eradicating bacteria and their byproducts from the walls of the root canal system. This mechanism is especially potent against Gram-positive bacteria, which possess cell walls surrounded by a delicate protein layer prone to degradation at higher temperatures [37]. Consequently, this phenomenon could potentially account for the significant bacterial reduction observed across all three groups employing ultrasonic activation.

5% Sodium thiosulfate was used after irrigation with sodium hypochlorite as a neutralizer to remove ion traces of the studied solution so as not to affect the result of the bacterial culture and give false negative results [38]. Additionally, sodium thiosulfate solution is reported as an antioxidant and is bioacceptable [39]. However, copious irrigation with distilled water was adopted to remove traces of the QROXB2 photosensitizer, as its neutralizer (Dey-Engley neutralizing broth) has never been used before in clinical studies within the context of endodontic therapy in patients and has never been reported to be bioacceptable.

Bacterial samples were accomplished using sterile paper points and a sterile hedströem file which used in an in-and-out motion along the root canal to obtain a more realistic bacterial sample because sampling with only paper points has limitations and the reason is that these samples can be taken only from microorganisms present in the root canal space, while those inside the dentine tubules cannot be detected [40].

The bacterial culture method for samples was used as it is an easy and reliable method to perform [41]. Moreover, it was adopted in recent studies [24, 42].

Sodium hypochlorite 5.25% was used as a final irrigation protocol alongside EDTA 17% to achieve the best photosensitizer and Ca(OH)_2_ dressing debris removal in the cervical, middle, and apical regions of the root canal [43, 44].

It is worth noting that the aim of this study was not to evaluate the effect of mechanical root canal preparation on reducing bacterial count present in necrotic canals. Since all endodontic preparation procedures were standardized across all samples, the impact of this factor was ignored.

Minhaco and colleagues [17] used aPDT to reduce the bacterial count of bacterial biofilms, where blue light with a wavelength of 450 nm was used to activate a mixture of curcumin and ultra-small particles of glycolic acid. As a photosensitizer at different concentrations and with a pre-irradiation time of 5 min, it led to a statistically significant reduction in the bacterial number in biofilms for different types of bacteria.

Also, Afrasiabi and colleagues [18] activated Riboflavin (vit B2) with 450 nm wavelength blue light after applying it to *Streptococcus faecalis* cultured in brain heart infusion (BHI). The study showed that increasing both the exposure time and the concentration of riboflavin increases the ability of this technique to eliminate bacterial count. But even at the highest concentrations, it is still unable to eliminate bacteria as effectively as sodium hypochlorite.

In this study, all three disinfecting techniques reduced the count of colony-forming units (CFU) and showed high antibacterial activity in necrotic root canals.

aPDT showed no statistically significant difference when compared to passive ultrasonic irrigation but showed higher and more promising results when compared to Ca(OH)2 dressings. This result can be explained by the idea that even though calcium hydroxide can penetrate to depths of 200 microns in dentine tubules, it is ineffective against biofilms [45]. It has been reported that Ca(OH)2 cannot disintegrate the cytoplasmic membranes and produce reactive oxygen species so it can not destroy the biofilm structure [46].

In addition, it’s worth noting that calcium hydroxide dressing induced this reduction in 7 days, aPDT and PUI could bring about this reduction in the colonies in only 5 min (the whole process) [47, 48].

These results were consistent with other studies, presenting significant differences between aPDT and Ca(OH)2 dressings in bacterial count reduction such as the findings concluded by Asnaashari and colleagues [47, 49], who used visible red light of (660 nm, 635 nm wavelength respectively) and (methylene blue, toluidine blue photosensitizer, respectively) for aPDT technique, and Devaraj and colleagues [45] who used curcumin as a photosensitizer activated by 380 nm wavelength blue light. Ca(OH)2 group in both studies had the weakest effect on reducing the bacterial count in the necrotic root canals.

On the other hand, Ahangari and colleagues [50] used Methylene blue as a photosensitizer with 810 nm wavelength red light in one session and calcium hydroxide dressing for 1 week and found that there were no statistically significant differences between the two study groups. This difference may be because Ahangary and colleagues’ study was performed on retreatment cases, unlike this study, which was performed in cases with primary periapical infections. The difference may also be explained by using different light sources and photosensitizers.

Rabello and colleagues [19] found that using Ca(OH)2 dressing for one week followed by aPDT resulted in the maximum bacterial count reduction compared with either of the two techniques when used alone. The results of this study are also consistent with the results of Miranda and colleagues [51], which found similar antibacterial activity of aPDT and PUI in extracted teeth infected with *Streptococcus* bacteria.

However, Niavarzi and colleagues [52] compared (aPDT using 660 nm wavelength red light and methylene blue photosensitizer- PUI- aPDT+PUI). This study found that activating irrigation with ultrasound led to reducing the bacterial count more effectively than the aPDT technique and that the combination of the two techniques gave the best results. This contradiction can be explained by using different wavelengths and photosensitizers. It can also be explained by the fact that Niavarzi studied the reduction of the bacterial count of Enterococcus faecalis only.

The current results also differed from the results of Ahangari who used a light with a 630 nm wavelength light source of toluidine blue photosensitizer and found the superiority of the PUI technique over the aPDT technique in reducing the bacterial number in necrotic root canals.

Beus and colleagues [35] found no significant difference in bacterial count reduction when comparing PUI and Ca(OH)2 dressings. Both Beus and colleagues and Carvalho and colleagues [53] indicated that using PUI before applying Ca(OH)2 dressings improved the antibacterial activity of root canal treatment.

These studies may differ in findings due to the differences in teeth treatment and irrigation protocols used in each study.

The main limitation of using aPDT is teeth discoloration as a possible side effect but applying photosensitizer in limited pre-irradiation time reduced this effect to its minimum [54]. Also using 2.5% sodium hypochlorite in final irrigation was effective in preventing tooth staining associated with the application of photosensitizer [55].

Further clinical and microbiological studies are needed to enhance the understanding of the treatment behavior with the adjunct of aPDT. This includes conducting similar studies on teeth with more complex root canal systems or using other natural photosensitizers. Additionally, conducting the same study in a laboratory setting to evaluate the effect of this protocol on each type of bacterial strain proposed to be present in necrotic canals with large periapical lesions would be beneficial.

## Conclusion

Antimicrobial Photodynamic Therapy (aPDT) can be considered an adjunct technique to passive ultrasonic irrigation and calcium hydroxide dressings, where it showed effectiveness in bacterial count reduction similar to PUI and higher than Ca(OH)_2_ dressings.

## Data Availability

De-identified data are available upon reasonable request to the corresponding author.
